# Quercetina Melhora o Perfil Lipídico e Apolipoproteico em Ratos Tratados com Glicocorticóides em Altas Doses

**DOI:** 10.36660/abc.20180397

**Published:** 2020-07-28

**Authors:** Hoda Derakhshanian, Mahmoud Djalali, Abolghassem Djazayery, Mohammad Hassan Javanbakht, Mahnaz Zarei, Azita Hekmatdoost, Ghazaleh Eslamian, Seyyedeh Somayyeh Mirhashemi, Ahmad Reza Dehpour

**Affiliations:** 1 Probiotic Research Center Alborz University of Medical Sciences Karaj Irã Dietary Supplements and Probiotic Research Center, Alborz University of Medical Sciences, Karaj - Irã; 2 Department of Biochemistry Nutrition and Genetics School of Medicine Alborz University of Medical Sciences Karaj Irã Department of Biochemistry Nutrition and Genetics, School of Medicine, Alborz University of Medical Sciences, Karaj - Irã; 3 Department of Cellular and Molecular Nutrition School of Nutritional Sciences and Dietetics Tehran University of Medical Sciences Tehran Irã Department of Cellular and Molecular Nutrition, School of Nutritional Sciences and Dietetics, Tehran University of Medical Sciences, Tehran - Irã; 4 Department of Community Nutrition School of Nutritional Sciences and Dietetics Tehran University of Medical Sciences Tehran Irã Department of Community Nutrition, School of Nutritional Sciences and Dietetics, Tehran University of Medical Sciences, Tehran - Irã; 5 Faculty of Nutrition Sciences and Food Technology National Nutrition and Food Technology Research Institute Shahid Beheshti University of Medical Sciences Tehran Irã Department of Clinical Nutrition and Dietetics, Faculty of Nutrition Sciences and Food Technology, National Nutrition and Food Technology Research Institute, Shahid Beheshti University of Medical Sciences, Tehran - Irã; 6 Student Research Committee Shahid Beheshti University of Medical Sciences Tehran Iran Student Research Committee, Shahid Beheshti University of Medical Sciences, Tehran - Iran; 7 Department of Pharmacology School of Medicine Tehran University of Medical Sciences Tehran Irã Department of Pharmacology, School of Medicine, Tehran University of Medical Sciences, Tehran - Irã; 8 Experimental Medicine Research Center Tehran University of Medical Sciences Tehran Irã Experimental Medicine Research Center, Tehran University of Medical Sciences, Tehran, Irã.

**Keywords:** Ratos Sprague-Dawley, Anti-inflamatórios, Queraracina, Glicocorticoides, Dislipidemias, Triglicérides, Colesterol

## Abstract

**Fundamento:**

Os glicocorticóides (GCs) são amplamente prescritos para o tratamento de numerosos distúrbios clínicos devido às suas propriedades anti-inflamatórias e imunomoduladoras, e um dos efeitos indesejáveis mais comuns desses medicamentos é a dislipidemia.

**Objetivo:**

Avaliar o efeito da quercetina, um flavonoide derivado de plantas, no perfil lipídico de ratos tratados com glicocorticóides em altas doses.

**Métodos:**

Um total de 32 ratos *Sprague-Dawley* foram distribuídos aleatoriamente entre quatro grupos (8 ratos por grupo) e tratados por 6 semanas com uma das seguintes opções : (i) solução salina normal; (ii) 40 mg/kg de succinato sódico de metilprednisolona (MP); (iii) MP + 50 mg/kg de quercetina; (iv) MP + 150 mg/kg de quercetina. O MP foi injetado por via subcutânea e a quercetina foi administrada por gavagem oral 3 dias por semana. No final do estudo, o perfil lipídico dos animais foi medido através de kits enzimáticos. Os dados foram analisados e a significância estatística foi estabelecida em p <0,05.

**Resultados:**

Os níveis séricos médios de colesterol total (CT), triglicerídeos (TG) e LDL aumentaram drasticamente em animais tratados com GC em comparação com o grupo controle. Ambas as doses de quercetina (50 e 150 mg/kg) melhoraram o CT (43% e 45%), LDL (56% e 56%) e TG (46% e 55%, respectivamente). A razão Apo B/A1 diminuiu mais de 20% após a ingestão de Anti-Inflamatory Agents.

**Conclusões:**

Esses dados sugerem que a ingestão de quercetina Quercetin; induzida por glicocorticóides. (Arq Bras Cardiol. 2020; 115(1):102-108)

## Introdução

Glicocorticoides como prednisona, metilprednisolona e dexametasona são amplamente prescritos para o tratamento de numerosas doenças clínicas, incluindo doenças pulmonares, gastrointestinais, hematológicas, cutâneas e renais, bem como transplantes de órgãos, principalmente devido às suas propriedades anti-inflamatórias e imunomoduladoras.^1^ Embora esses medicamentos tenham tais benefícios, seus efeitos adversos, como hiperglicemia, hipertensão, hiperlipidemia, osteoporose, atrofia muscular e obesidade, devem ser levados a sério.^2^ O metabolismo lipídico deficiente é uma das reações indesejáveis mais comuns; a utilização de GCs em altas doses ou administrados em longo prazo causam nos usuários uma aparência semelhante à da síndrome de Cushing. Em outras palavras, a hipercolesterolemia e a hipertrigliceridemia são altamente prevalentes em pacientes submetidos a terapia com GC por períodos prolongados e, em última análise, podem levar a riscos de aterosclerose.^3 - 4^ No entanto, quando a administração desses medicamentos imunossupressores é inevitável, devemos procurar alguns medicamentos ou produtos naturais para minimizar seus efeitos indesejáveis.

A quercetina, 3,3’,4’,5,7-penta-hidroxiflavona, 2-(3,4-di-hidroxifenil)-3,5,7-tri-hidroxi-4H-cromen-4-ona, C15H10O7, é um flavonoide derivado de planta, isolado de cebolas, maçãs, uvas, vegetais folhosos e chá;^5 , 6^ Esse composto de polifenol de ocorrência natural é geralmente conhecido por suas propriedades antioxidantes e anti-inflamatórias e foi relatado como sendo um fator de melhora do sistema de defesa antioxidante, diminuindo a incidência de doenças cardiovasculares, neoplásicas e inflamatórias.^7 - 9^ Como o equilíbrio oxidante-antioxidante e o status da inflamação desempenham um papel importante na etiologia de muitas doenças, os compostos flavonoides têm sido destacados como agentes preventivos ou terapêuticos naturais^10 , 11^ . Além disso, alguns estudos anteriores relataram impacto benéfico da quercetina na síndrome metabólica e no metabolismo lipídico.^12 , 13^ O objetivo deste estudo é avaliar o efeito da quercetina no perfil lipídico de ratos tratados com glicocorticoide em altas doses.

## Materiais e métodos

### Animais

Um total de 32 ratos *Sprague-Dawley* com idades entre 6 e 7 meses, pesando 210 ± 30 gramas, foram obtidos do Razi Institute (Karaj, Irã). Os animais foram aclimatados às condições laboratoriais padrão (temperatura 20-25 °C e ciclo de 12 horas claro/escuro) por 10 dias antes do início do experimento principal. Foram fornecidas *ad libitum* água limpa e dieta padrão de ração peletizada (Danbehparvar, Thran, Irã). O protocolo experimental estava de acordo com os *Principles of Laboratory Animal Care.* ^14^ O tamanho da amostra foi calculado com 80% de potência, utilizando um teste bilateral com nível de significância de 5% e com base no tamanho do efeito de 0,5.

### Produtos químicos

Succinato sódico de metilprednisolona (MP) foi utilizado como glicocorticoide (SOLU-MEDROL, Pfizer Pharmaceuticals, NY, EUA) para gerar dislipidemia induzida por GC.^15^ A quercetina, com uma pureza de 95%, foi obtida da Sigma-Aldrich Chemicals (St. Louis, MO, EUA) e a suspensão de quercetina foi preparada adicionando-se quercetina à solução aquosa de carboximetilcelulose (CMC) a 0,05%, imediatamente antes de ser administrada por gavagem oral.

### Procedimento experimental

Trinta e dois animais foram distribuídos aleatoriamente em quatro grupos, utilizando o esquema de randomização em bloco. Cada grupo experimental continha oito ratos que foram tratados por seis semanas. Todos os grupos foram injetados por via subcutânea (s.c.) com MP (40 mg/kg de peso corporal), exceto o grupo controle que recebeu solução salina normal três dias por semana. Cada um dos três grupos injetados com glicocorticoide recebeu um dos seguintes tratamentos: CMC como placebo, 50 mg/kg de quercetina ou 150 mg/kg de quercetina. Todos os tratamentos foram administrados três dias por semana, por via oral. Ao final do estudo, todos os animais foram anestesiados com injeção intraperitoneal (i.p.) de cetamina juntamente com xilazina (50 mg/kg e 30 mg/kg, respectivamente).^15 , 16^ As amostras de sangue foram coletadas por punção cardíaca e imediatamente centrifugadas a 3000 rpm por 10 min para isolamento sérico e armazenadas a -80°C até a análise do perfil lipídico. Os ratos foram submetidos a jejum por 12 a 14 horas e todas as amostras de sangue foram coletadas entre 8 e 10 horas da manhã. Foram utilizados kits enzimáticos disponíveis no mercado para medir as concentrações séricas de colesterol total (CT), lipoproteína de alta densidade (HDL, *high-density lipoprotein* ) e triglicérides (TG) em duplicata (Pars Azmoon Co., Teerã, Irã), e Apo A e Apo B foram medidos através de métodos imunoturbidimétricos (biorexfars LTD, Irã). O nível de lipoproteína de baixa densidade (LDL, *low-density lipoprotein* ) foi calculado utilizando-se a equação de Friedewald.^17^ Os animais foram pesados no início e ao final do estudo.

### Análise estatística

Todos os dados foram apresentados como média ± desvio padrão (DP) e analisados pelo *Statistical Package for Social Sciences* (versão 23.0; SPSS Inc., Chicago, EUA). O teste de Kolmogorov-Smirnov foi utilizado para avaliar a normalidade dos dados. As diferenças estatísticas entre os grupos foram avaliadas através da análise de variância (ANOVA One-Way) seguida pelo teste *post hoc* de Bonferroni. A significância estatística foi estabelecida em p<0,05.

## Resultados

Embora o peso corporal médio dos ratos tenha sido o mesmo em todos os grupos no início do experimento, após seis semanas de intervenção, todos os animais tratados com glicocorticóides apresentaram uma redução significativa de peso em comparação com seus próprios pesos iniciais e com seus controles pareados por idade ( Tabela 1 ).

**Tabela 1:** – Peso corporal inicial e final (gramas) dos grupos experimentais

Peso corporal	Controle	MP	MP+Q50	MP+Q150
Inicial	212±29	212±27	210±28	212±28
Final	214±30^†^	182±22^*,‡^	185±20^*,‡^	180±16^*,‡^

Os dados são apresentados como Média ± DP. N = 8 para todos os grupos. MP: metilprednisolona; Q50: quercetina 50 mg/kg; Q150: quercetina 150 mg/kg; Análise de variância (ANOVA) seguida pelo teste de Bonferroni. ^*^p <0,05 comparado ao grupo controle, ^†^p <0,05 comparado ao grupo MP, ^‡^p <0,05 comparado ao peso inicial do mesmo grupo.

**Figura 1:**
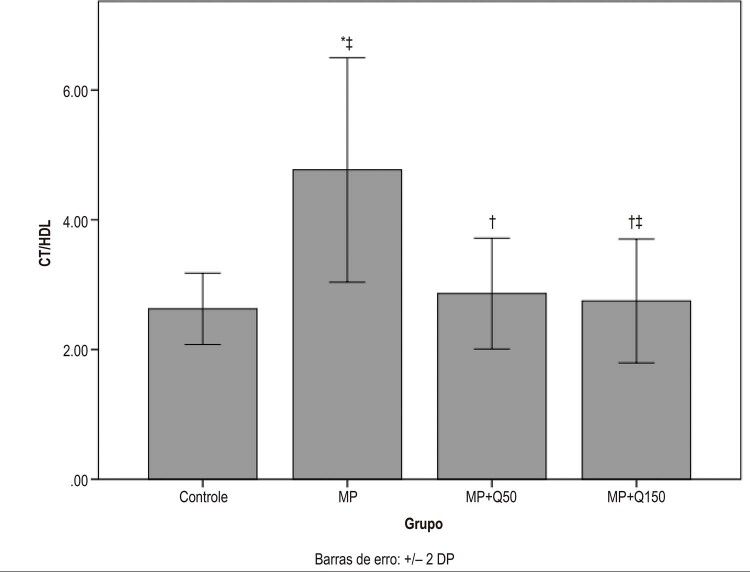
– Média da razão colesterol total/HDL em diferentes grupos. Dados apresentados como Média ± DP. N = 8 para todos os grupos. MP: metilprednisolona; Q50: quercetina 50 mg/kg; Q150: quercetina 150 mg/kg; CT: colesterol total; HDL: lipoproteína de alta densidade; *p<0,05 comparado ao grupo controle, ^†^p<0,05 comparado ao grupo MP, ^‡^p<0,05 comparado ao MP + Q50.

**Figura 2:**
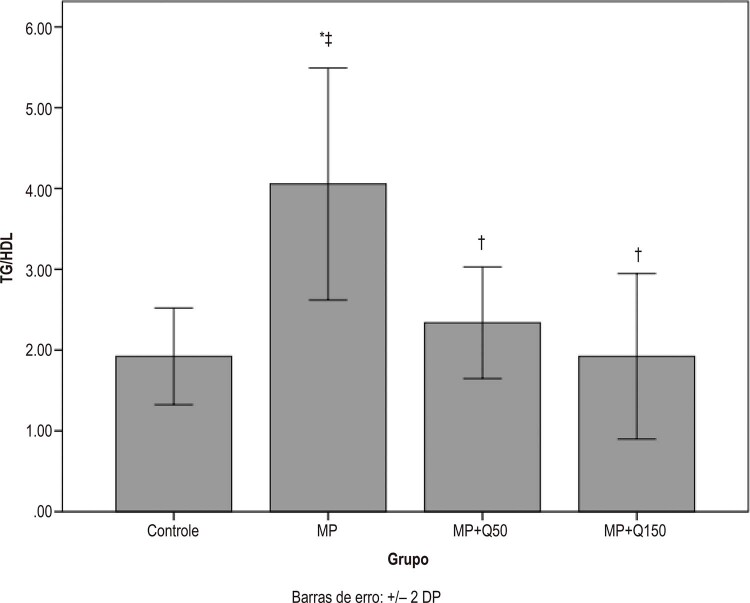
– Média da razão triglicerídeos para HDL em diferentes grupos. Dados apresentados como Média ± EP. n = 8 para todos os grupos. MP: metilprednisolona; Q50: quercetina 50 mg/kg; Q150: quercetina 150 mg/kg; TG: triglicerídeos; HDL: lipoproteína de alta densidade; ^*^p<0,05 comparado ao grupo controle, ^†^p<0,05 comparado ao grupo MP, ^‡^p<0,05 comparado ao MP + Q50.

**Figura 3:**
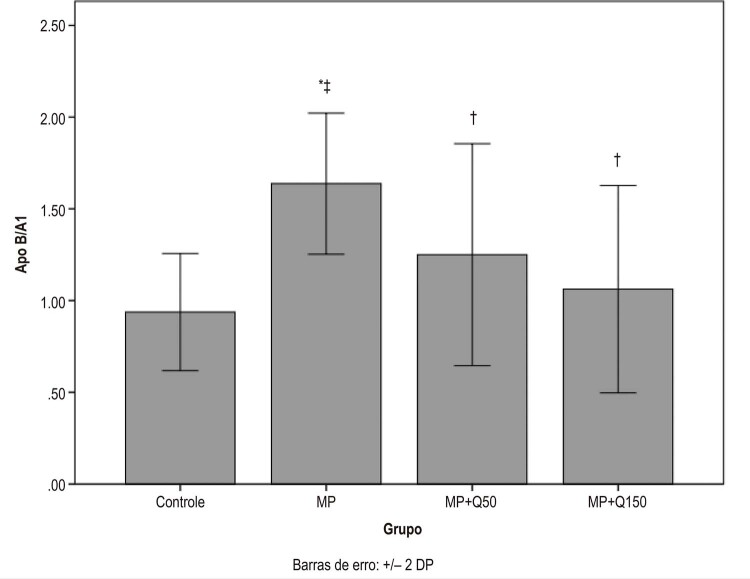
– Média da razão de Apolipoproteína B para Apolipoproteína Al em diferentes grupos. Dados apresentados como Média ± EP. n = 8 para todos os grupos. MP: metilprednisolona; Q50: quercetina 50 mg/kg; Q150: quercetina 150 mg/kg; ApoB/Al: razão Apolipoproteína B para Apolipoproteína Al; ^*^p<0,05 comparado ao grupo controle, ^†^p<0,05 comparado ao grupo MP, ^‡^p<0,05 comparado ao MP + Q50.

Após seis semanas de injeção de metilprednisolona, os níveis médios de colesterol e triglicérides no plasma aumentaram drasticamente em animais tratados com glicocorticoides, em comparação com o grupo controle. Ambas as doses de quercetina (50 e 150 mg/kg) melhoraram a hipercolesterolemia e a hipertrigliceridemia em comparação com o grupo MP, e a mesma tendência foi observada nos níveis de LDL. Além disso, a injeção de MP causou um aumento moderado nos níveis de HDL, os quais não sofreram alteração significativa após a suplementação com quercetina. No entanto, a redução das razões CT/HDL, TG/HDL e LDL/HDL foi estatisticamente e clinicamente significativa. Além disso, a razão Apo B/A1 diminuiu mais de 20% após a ingestão de quercetina ( Tabela 2 ; Figuras 1-3 ). Parece que uma dose mais alta de quercetina não possui superioridade visível para a melhora do colesterol e apolipoproteínas. No entanto, foi encontrada uma correlação negativa entre a dose de quercetina e TG, bem como a razão CT/HDL (-0,87 e -0,75, respectivamente).

**Tabela 2:** – Perfil lipídico dos grupos experimentais após seis semanas de intervenção

	Controle	MP	MP+Q50	MP+Q150	Valor de p
CT (mg/dl)	89,12±3,35	193,50±12,77^*,‡^	108,75±15,47^*,†^	105,87±11,25^*,†^	<0,001
HDL (mg/dl)	34,25±3,69	41,37±5,75^*^	38,25±4,77	39,00±4,07	=0,03
LDL (mg/dl)	41,87±3,79	119,22±12,70^*,‡^	52,72±15,15^†^	52,22±10,87^†^	<0,001
TG (mg/dl)	65,00±4,34	164,50±9,36^*,‡^	88,87±12,93^*,†^	73,25±11,33^†,‡^	<0,001
CT/HDL	2,62±0,27	4,76±0,86^*,‡^	2,86±0,42^†^	2,74±0,47^†,‡^	<0,001
TG/HDL	1,92±0,29	4,05±0,71^*,‡^	2,33±0,34^†^	1,92±0,51^†^	<0,001
LDL/HDL	1,24±0,23	2,95±0,73^*,‡^	1,39±0,43^†^	1,36±0,38^†^	<0,001
Apo B/ Al	0,93±0,16	1,63±0,19^*,‡^	1,25±0,30^†^	1,06±0,28^†^	<0,001

Os dados são apresentados como Média ± DP. n = 8 para todos os grupos. MP: metilprednisolona; Q50: quercetina 50 mg/kg; Q150: quercetina 150 mg/kg; CT: colesterol total; TG: triglicerídeos; HDL: lipoproteína de alta densidade; LDL: lipoproteína de baixa densidade; ApoB/Al: razão Apolipoproteína B para Apolipoproteína Al; Análise de variância (ANOVA) seguida pelo teste de Bonferroni. ^*^p <0,05 comparado ao grupo controle, ^†^p <0,05 comparado ao grupo MP, ^‡^p <0,05 comparado ao MP + Q50.

## Discussão

Nossos resultados revelaram que a administração de altas doses de glicocorticoide por 6 semanas aumentou drasticamente a concentração sérica de colesterol total, LDL e triglicérides. No entanto, a suplementação oral com duas doses diferentes de quercetina, como uma flavona de ocorrência natural tendo sido anteriormente relatada como benéfica na síndrome metabólica, reverteu de maneira evidente os efeitos indesejáveis da metilprednisolona. Foram escolhidas diferentes doses de quercetina, uma vez que a dose mais baixa pode ser fornecida por uma dieta rica em quercetina e a mais alta pode ser conseguida com a utilização de suplementos disponíveis comercialmente.^18^ Desnecessário dizer que a diferente taxa metabólica de ratos e humanos foi levada em consideração para a determinação da dose.^19^ Os resultados finais indicaram que 150 mg/kg de quercetina não foram muito mais eficazes que 50 mg/kg para melhorar o perfil lipídico, exceto pela concentração de TG, que desceu ao nível dos controles como resultado da administração de doses elevadas de quercetina. A metilprednisolona também causou um aumento moderado no nível de HDL, o qual não foi alterado significativamente após a suplementação com quercetina.

Embora o impacto hiperlipidêmico dos GCs tenha sido observado nas últimas décadas, os mecanismos moleculares ainda não são bem reconhecidos. Alguns estudos *in vitro* e *in vivo* demonstraram que esses fármacos anti-inflamatórios podem aumentar diretamente a produção hepática de HDL, up-regular a atividade da lipoproteína lipase e prejudicar o catabolismo do LDL, reduzindo a expressão e a atividade dos receptores hepáticos de LDL.^15 , 20^ Consequentemente, contribuem para o desenvolvimento de fígado gorduroso, aumentando a síntese de ácidos graxos e diminuindo a β-oxidação.^21^

Por outro lado, os flavonoides têm sido descritos como moduladores do metabolismo lipídico. Eles atuam principalmente através da inibição da fosfodiesterase, alteração da absorção hepática de colesterol e produção e secreção de triglicérides.^22 - 25^ Além disso, a quercetina, como um potente antioxidante distribuído tanto na bicamada lipídica quanto na fase aquosa da célula, pode suprimir a peroxidação lipídica pela atividade de eliminação de radical.^26^ Grandes estudos demonstraram que a razão Apo B/Al é superior ao colesterol total e TG para prever o risco cardiovascular em ambos os sexos e em todas as faixas etárias.^27^ Considerando que a razão Apo B/Al é uma medida do número de partículas aterogênicas de Apo B sobre o número de partículas anti-aterogênicas de Apo Al, existe também a possibilidade de a mesma ser um fator mais importante que a quantidade de lipídios transportada por partícula. No presente estudo, a ingestão de quercetina diminuiu significativamente a razão Apo B/AI, o que pode ser um importante indicador de menor risco cardiovascular no futuro.^27 , 28^

Ao final da intervenção, todos os animais tratados com glicocorticoides apresentaram uma redução significativa de peso em comparação aos seus controles, o que pode ter sido causado por anorexia induzida por glicocorticoides nos ratos, relatada anteriormente,^29^ ou por proteólise e perda muscular graves.^30^ Uma das limitações deste estudo foi a falta de dados precisos sobre a ingestão alimentar dos animais, o que poderia ser muito útil para a interpretação da perda de peso induzida por GC nos ratos. No geral, nossos achados estão de acordo com estudos anteriores que relataram efeitos benéficos dos flavonoides no metabolismo lipídico.^31^ Esta é a primeira pesquisa que avalia o impacto da quercetina na hiperlipidemia induzida por GC. No entanto, o efeito hipolipidêmico de alguns outros flavonoides foi relatado em ratos tratados com GC.^32^ Outras propriedades favoráveis da quercetina na melhora da densidade óssea e na modificação da glicemia tornam esse flavonoide uma excelente opção para controlar os efeitos colaterais dos glicocorticoides.^33^

## Conclusão

A administração de quercetina, em doses de 50 e 150 mg/kg, pode reverter os efeitos indesejáveis de altas doses de glicocorticoides no perfil lipídico de ratos e pode ser considerada para terapia combinada com GCs para minimizar a dislipidemia resultante.
