# Supplementary material for: Quercetina Melhora o Perfil Lipídico e Apolipoproteico em Ratos Tratados com Glicocorticóides em Altas Doses
Source: Arq Bras Cardiol. 2020 Jul 28;115(1):102–8. [Article in Portuguese] doi: 10.36660/abc.20180397 (PMC8384335; doi:10.36660/abc.20180397)
Supplement: Translation

# Translation: Quercetin Ameliorates Lipid and Apolipoprotein Profile in High-Dose Glucocorticoid Treated Rats

**Authors:** Hoda Derakhshanian, Mahmoud Djalali, Abolghassem Djazayery, Mohammad Hassan Javanbakht, Mahnaz Zarei, Azita Hekmatdoost, Ghazaleh Eslamian, Seyyedeh Somayyeh Mirhashemi, Ahmad Reza Dehpour

**Affiliations:** 1 Irã Dietary Supplements and Probiotic Research Center, Alborz University of Medical Sciences, Karaj - Irã; 2 Irã Department of Biochemistry Nutrition and Genetics, School of Medicine, Alborz University of Medical Sciences, Karaj - Irã; 3 Irã Department of Cellular and Molecular Nutrition, School of Nutritional Sciences and Dietetics, Tehran University of Medical Sciences, Tehran - Irã; 4 Irã Department of Community Nutrition, School of Nutritional Sciences and Dietetics, Tehran University of Medical Sciences, Tehran - Irã; 5 Irã Department of Clinical Nutrition and Dietetics, Faculty of Nutrition Sciences and Food Technology, National Nutrition and Food Technology Research Institute, Shahid Beheshti University of Medical Sciences, Tehran - Irã; 6 Iran Student Research Committee, Shahid Beheshti University of Medical Sciences, Tehran - Iran; 7 Irã Department of Pharmacology, School of Medicine, Tehran University of Medical Sciences, Tehran - Irã; 8 Irã Experimental Medicine Research Center, Tehran University of Medical Sciences, Tehran, Irã.

**Keywords:** Rats, Sprague-Dawley, Anti-Inflammatory Agents, Quercetin, Glucocorticoids, Dyslipidemias, Triglycerides, Cholesterol

## Abstract

**Background:**

Glucocorticoids (GCs) are widely prescribed for the treatment of numerous clinical disorders due to their anti-inflammatory and immune-modulatory properties and one of the most common untoward effects of these drugs is dyslipidemia.

**Objective:**

To evaluate the effect of quercetin, a plant-derived flavonoid, on the lipid profile of high-dose glucocorticoid treated rats.

**Methods:**

A total of 32 Sprague-Dawley rats, were randomly distributed among four groups (8 rats per group) and treated for 6 weeks with one of the following: (i) normal saline; (ii) 40 mg/kg methylprednisolone sodium succinate (MP); (iii) MP + 50 mg/kg quercetin; (iv) MP + 150 mg/kg quercetin. MP was injected subcutaneously, and quercetin was administered by oral gavage 3 days a week. At the end of the study, the animals’ lipid profile was measured by enzymatic kits. Data were analyzed and statistical significance was set at p<0.05.

**Results:**

The mean serum total cholesterol (TC), triglyceride (TG) and LDL levels were drastically increased in GC-treated animals compared with the control group. Both doses of quercetin (50 and 150 mg/kg) ameliorated TC (43% and 45%), LDL (56% and 56%) and TG (46% and 55% respectively). Apo B/A1 ratio decreased more than 20% following quercetin intake and the decline in TC/HDL, TG/HL, LDL/HDL ratios were significant.

**Conclusions:**

These data suggest that quercetin intake with both doses of 50 and 150 mg/kg could be considered as a protective agent for glucocorticoid-induced dyslipidemia. (Arq Bras Cardiol. 2020; 115(1):102-108.)

## Introduction

Glucocorticoids such as prednisone, methylprednisolone, and dexamethasone are widely prescribed for the treatment of numerous clinical disorders, including pulmonary, gastrointestinal, hematological, skin, and renal diseases, as well as organ transplants, particularly due to their anti-inflammatory and immune-modulatory properties.^1^ Although these drugs have such benefits, their adverse effects such as hyperglycemia, hypertension, hyperlipidemia, osteoporosis, muscle atrophy and obesity must be taken seriously.^2^ Impaired lipid metabolism, as one of the most common undesirable reactions, in high-dose or long-term GC users, resembles Cushing’s syndrome. In other words, hypercholesterolemia and hypertriglyceridemia are highly prevalent in patients undergoing GC therapy for prolonged periods and may ultimately lead to risks for atherosclerosis.^3 - 4^ However, when the administration of these immunosuppressive drugs is inevitable, one should look for some drugs or natural products to minimize their untoward effects.

Quercetin, 3,3’,4’,5,7-Pentahydroxyflavone, 2-(3,4-Dihydroxyphenyl)-3,5,7-trihydroxy-4H-chromen-4-one, C15H10O7, is a plant-derived flavonoid, isolated from onions, apples, grapes, leafy vegetables and tea.^5 , 6^ This naturally occurring polyphenol compound is generally known for its antioxidant and anti-inflammatory properties and is reported to enhance the antioxidant defense system, and decrease the incidence of cardiovascular, neoplastic and inflammatory diseases.^7 - 9^ Since the oxidant-antioxidant balance and inflammation status play an important role the etiology of many diseases, flavonoid compounds have been in the spotlight as natural preventive or therapeutic agents.^10 , 11^ In addition, some previous studies reported the beneficial impact of quercetin on metabolic syndrome and lipid metabolism.^12 , 13^ The aim of this study is to evaluate the effect of quercetin on lipid profile of rats treated with high-dose glucocorticoid.

## Materials and methods

### Animals

A total of 32 Sprague-Dawley rats, aged 6-7 months, weighing 210±30 grams were obtained from the Razi Institute (Karaj, Iran). The animals were acclimatized to the standard laboratory conditions (temperature 20-25˚C, and a 12-h light/dark cycle) for 10 days before the beginning of the main experiment. Clean water and pelleted standard chow diet (Danbehparvar, Thran, Iran) were provided *ad libitum* . The experimental protocol was in accordance with the Principles of Laboratory Animal Care.^14^ The sample size was calculated with 80% power, using a two-sided test at the 5% significance level and based on the effect size of 0.5.

### Chemicals

Methylprednisolone sodium succinate (MP) was used as the glucocorticoid (SOLU-MEDROL, Pfizer Pharmaceuticals, NY, U.S.A) for generating GC-induced dyslipidemia.^15^ Quercetin, with a purity of 95%, was obtained from Sigma-Aldrich Chemicals (St. Louis, MO, U.S.A) and the quercetin suspension was prepared by adding quercetin to 0.05% aqueous carboxymethyl cellulose (CMC) solution immediately before being administered by oral gavage.

### Experimental procedure

Thirty-two animals were randomly distributed into four groups, using the block randomization scheme. Each experimental group contained eight rats, which were treated for six weeks. All groups were injected subcutaneously (s.c.) with MP (40 mg/kg body weight), except the control group, which received normal saline solution three days a week. Each of the three glucocorticoid-injected groups received one of the following treatments: CMC as placebo, 50 mg/kg quercetin or 150 mg/kg quercetin. All treatments were given three days a week *per os* . At the end of the study all animals were anesthetized with an intra-peritoneal (i.p.) injection of ketamine together with xylazine (50 mg/kg and 30 mg/kg respectively).^15 , 16^ Blood samples were collected by cardiac puncture and were immediately centrifuged at 3000 rpm for 10 min for serum isolation and stored at -80˚C until analysis of the lipid profile. The rats were fasted for 12-14 hours and all blood samples were collected between 8 and 10 am. Commercially available enzymatic kits were used to measure the serum concentrations of total cholesterol (TC), high density lipoprotein (HDL), and triglycerides (TG) in duplicate tests (Pars Azmoon Co., Tehran, Iran) and Apo A and Apo B were measured by immunoturbidimetric methods (biorexfars LTD, Iran). Low-density lipoprotein (LDL) level was calculated using the Friedewald equation.^17^ Animals were weighed at the beginning and end of the study.

### Statistical analysis

All data were presented as mean ± standard deviation (SD) and analyzed by the Statistical Package for Social Sciences (version 23.0; SPSS Inc., Chicago, USA). The Kolmogorov-Smirnov test was used to assess the normality of the data. Statistical differences between groups were evaluated using analysis of variance (one-way ANOVA) followed by Bonferroni *post hoc* test. Statistical significance was set at p *<* 0.05.

## Results

Although the average body weight of rats was the same in all groups at the beginning of the experiment, after six weeks of intervention, all glucocorticoid-treated animals showed a significant weight reduction compared with their own initial weights and with their age-matched controls ( Table 1 ).

**Table 1:** – Initial and final body weight (gram) of experimental groups

Body weight	Control	MP	MP+Q50	MP+Q150
Initial	212±29	212±27	210±28	212±28
Final	214±30^†^	182±22^*,‡^	185±20^*,‡^	180±16^*,‡^

Data are presented as Mean±SD. N=8 for all groups. MP, methylprednisolone; Q50, quercetin 50 mg/kg; Q150, quercetin 150 mg/kg; Analysis of variance (ANOVA) followed by Bonferroni test. * p<0.05 compared with control group,^†^p<0.05 compared with MP group,^‡^p<0.05 compared with the initial weight of same group.

Following six weeks of methylprednisolone injection, the mean plasma cholesterol and triglyceride levels were drastically increased in glucocorticoid-treated animals compared with the control group. Both doses of quercetin (50 and150 mg/kg) improved the hypercholesterolemia and hypertriglyceridemia in comparison with the MP group, and the same trend was observed for LDL levels. In addition, the MP injection caused a moderate increase in HDL levels, which was not significantly changed following quercetin supplementation. However, the reduction in TC/HDL, TG/HDL and LDL/HDL ratios were statistically and clinically significant. Moreover, Apo B/A1 ratio decreased more than 20% following quercetin intake ( Table 2 ; Figures 1-3 ). It seems that a higher dose of quercetin does not have a conspicuous superiority for cholesterol and apolipoprotein level improvement. However, a negative correlation was found between the quercetin dose and TG, as well as TC/HDL (-0.87 and -0.75 respectively).

**Table 2:** – Lipid profile of experimental groups after six weeks of intervention

	Control	MP	MP+Q50	MP+Q150	p value
TC (mg/dl)	89.12±3.35	193.50±12.77^*.‡^	108.75±15.47^*.†^	105.87±11.25^*.†^	<0.001
HDL (mg/dl)	34.25±3.69	41.37±5.75^*^	38.25±4.77	39.00±4.07	=0.03
LDL (mg/dl)	41.87±3.79	119.22±12.70^*.‡^	52.72±15.15^†^	52.22±10.87^†^	<0.001
TG (mg/dl)	65.00±4.34	164.50±9.36^*.‡^	88.87±12.93^*.†^	73.25±11.33^†.‡^	<0.001
TC/HDL	2.62±0.27	4.76±0.86^*.‡^	2.86±0.42^†^	2.74±0.47^†.‡^	<0.001
TG/HDL	1.92±0.29	4.05±0.71^*.‡^	2.33±0.34^†^	1.92±0.51^†^	<0.001
LDL/HDL	1.24±0.23	2.95±0.73^*.‡^	1.39±0.43^†^	1.36±0.38^†^	<0.001
Apo B/ Al	0.93±0.16	1.63±0.19^*.‡^	1.25±0.30^†^	1.06±0.28^†^	<0.001

Data are presented as Mean±SD. n=8 for all groups. MP: methylprednisolone; Q50: quercetin 50 mg/kg; Q150: quercetin 150 mg/kg; TC: total cholesterol; TG: triglyceride; HDL: high-density lipoprotein; LDL: low-density lipoprotein; ApoB/Al: apolipoprotein B to apolipoprotein Al ratio; Analysis of variance (ANOVA) followed by Bonferroni test.^*^p<0.05 compared with control group,^†^p<0.05 compared with MP group,^‡^p<0.05 compared with MP+Q50.

**Figure 1:** – Mean of the total cholesterol to HDL ratio in the different groups. Data presented as Mean±SD. N=8 for all groups. MP: methylprednisolone; Q50: quercetin 50 mg/kg; Q150: quercetin 150 mg/kg; TC: total cholesterol; HDL: high-density lipoprotein;^*^p<0.05 compared with control group,^†^p<0.05 compared with MP group,^‡^p<0.05 compared with MP+Q50.

## Discussion

Our findings revealed that the administration of high-dose glucocorticoid for 6 weeks drastically increased serum concentrations of total cholesterol, LDL and triglycerides. However, oral supplementation with two different doses of quercetin, as a naturally occurring flavone that was previously reported to be beneficial in metabolic syndrome, conspicuously reversed the undesirable effects of methylprednisolone. Different doses of quercetin were chosen, since the lower one can be provided by a quercetin-rich diet and the higher one might be taken as commercially available supplements.^18^ Needless to say, the different metabolic rates of rats and humans were taken into account for dose determination.^19^ The final results indicated that 150 mg/kg quercetin were not much more effective than 50 mg/kg to improve lipid profile, except for TG concentrations, which decreased to the control level as a result of high dose quercetin administration. Methylprednisolone also caused a moderate increase in HDL levels, which was not significantly changed following quercetin supplementation.

Although the hyperlipidemic impact of GCs has been noticed for the last decades, the molecular mechanisms are not well recognized yet. Some *in vitro* and *in vivo* studies demonstrated that these anti-inflammatory drugs can directly increase hepatic HDL production, up-regulate lipoprotein lipase activity and impair LDL catabolism by reducing hepatic LDL receptors expression and activity.^15 , 20^ Consequently, they contribute to fatty liver development by increasing fatty acid synthesis and decreasing β oxidation.^21^

On the other hand, flavonoids have been described as lipid metabolism modulators. They mostly act through the inhibition of phosphodiesterase, alteration of hepatic cholesterol absorption and triglyceride production and secretion.^22 - 25^ In addition, quercetin as a potent antioxidant distributed in both the lipid bilayer and aqueous phase of the cell, can suppress lipid peroxidation by radical scavenging activity.^26^ Large studies have shown that ApoB/Al ratio is superior to the total cholesterol and TG for cardiovascular risk prediction in both genders and at all age ranges.^27^ Given that the ApoB/Al ratio is a measurement of the number of ApoB atherogenic particles over the number of ApoAl anti-atherogenic particles, there is also a possibility that it is a more important factor than the amount of lipids carried per particle. In the present study, quercetin intake significantly decreased ApoB/AI ratio, which might be an important indicator of lower cardiovascular risk in the future.^27 , 28^

At the end of intervention, all glucocorticoid-treated animals showed a significant weight reduction compared to their controls, which might be due to glucocorticoid-induced anorexia in rats, which has been previously reported,^29^ or to severe proteolysis and muscle loss.^30^ One of the limitations of this study was the lack of precise data about the animals’ food intake, which could be very useful for the interpretation of GC-induced weight loss in rats. Overall, our findings are in accordance with previous studies reporting the beneficial effects of flavonoids on lipid metabolism.^31^ This is the first research evaluating the impact of quercetin on GC-induced hyperlipidemia. However, the hypolipidemic effect of some other flavonoids has been reported in GC-treated rats.^32^ Other favorable properties of quercetin in improving bone density and modifying blood glucose, make this flavonoid an excellent choice to control glucocorticoid side effects.^33^

## Conclusion

Quercetin administration, at both doses of 50 and 150 mg/kg, was able to reverse the untoward effects of high-dose glucocorticoids on the lipid profile of rats, and might be considered for combination therapy with GCs to minimize the resulting dyslipidemia.

**Figure 2:** – Mean of triglycerides to HDL ratio in the different groups. Data presented as Mean±SE. n=8 for all groups. MP: methylprednisolone; Q50: quercetin 50 mg/kg; Q150: quercetin 150 mg/kg; TG; triglyceride; HDL: high-density lipoprotein;^*^p<0.05 compared with control group,^†^p<0.05 compared with MP group,^‡^p<0.05 compared with MP+Q50.

**Figure 3:** – Mean of apolipoprotein B to apolipoprotein Al ratio in the different groups. Data presented as Mean±SE. n=8 for all groups. MP: methylprednisolone; Q50: quercetin 50 mg/kg; Q150: quercetin 150 mg/kg; ApoB/Al: apolipoprotein B to apolipoprotein Al ratio;^*^p<0.05 compared with control group,^†^p<0.05 compared with MP group,^‡^p<0.05 compared with MP+Q50.
